# *Gloriosa superba* Mediated Synthesis of Platinum and Palladium Nanoparticles for Induction of Apoptosis in Breast Cancer

**DOI:** 10.1155/2018/4924186

**Published:** 2018-07-02

**Authors:** Shalaka S. Rokade, Komal A. Joshi, Ketakee Mahajan, Saniya Patil, Geetanjali Tomar, Dnyanesh S. Dubal, Vijay Singh Parihar, Rohini Kitture, Jayesh R. Bellare, Sougata Ghosh

**Affiliations:** ^1^Department of Microbiology, Modern College of Arts, Science and Commerce, Ganeshkhind, Pune 411016, India; ^2^Institute of Bioinformatics and Biotechnology, Savitribai Phule Pune University, Pune 411007, India; ^3^Indian Institute of Science, Education and Research, Pashan, Pune 411008, India; ^4^Department of Biomedical Sciences and Engineering, BioMediTech, Tampere University of Technology, Korkeakoulunkatu 10, 33720 Tampere, Finland; ^5^Department of Applied Physics, Defense Institute of Advanced Technology, Girinagar, Pune 411025, India; ^6^Department of Chemical Engineering, Indian Institute of Technology Bombay, Powai, Mumbai 400076, India; ^7^Department of Microbiology, School of Science, RK University, Kasturbadham, Rajkot 360020, India

## Abstract

Green chemistry approaches for designing therapeutically significant nanomedicine have gained considerable attention in the past decade. Herein, we report for the first time on anticancer potential of phytogenic platinum nanoparticles (PtNPs) and palladium nanoparticles (PdNPs) using a medicinal plant *Gloriosa superba* tuber extract (GSTE). The synthesis of the nanoparticles was completed within 5 hours at 100°C which was confirmed by development of dark brown and black colour for PtNPs and PdNPs, respectively, along with enhancement of the peak intensity in the UV-visible spectra. High-resolution transmission electron microscopy (HRTEM) showed that the monodispersed spherical nanoparticles were within a size range below 10 nm. Energy dispersive spectra (EDS) confirmed the elemental composition, while dynamic light scattering (DLS) helped to evaluate the hydrodynamic size of the particles. Anticancer activity against MCF-7 (human breast adenocarcinoma) cell lines was evaluated using MTT assay, flow cytometry, and confocal microscopy. PtNPs and PdNPs showed 49.65 ± 1.99% and 36.26 ± 0.91% of anticancer activity. Induction of apoptosis was most predominant in the underlying mechanism which was rationalized by externalization of phosphatidyl serine and membrane blebbing. These findings support the efficiency of phytogenic fabrication of nanoscale platinum and palladium drugs for management and therapy against breast cancer.

## 1. Introduction

Spectacular development in the field of nanotechnology has led to the fabrication of exotic nanostructures with attractive physicochemical and optoelectronic properties. Nanomaterials have got broad-spectrum therapeutic applications which include carbon-based nanostructures, semiconductor quantum dots, polymeric particles, metallic nanoparticles, and magnetic nanoparticles. However, flexibility to vary the properties like shape, size, composition, assembly, and encapsulation has made metallic nanoparticles most preferred over others for biomedical applications [1]. Platinum-based therapeutic drugs, notably cisplatin and carboplatin, are exploited in chemotherapy against cancer, while platinum nanoparticles (PtNPs) have gained attention only recently [2]. Similarly, palladium nanoparticles (PdNPs) are also reported to exhibit anticancer activity against human leukemia (MOLT-4) cells [3]. Although there are so many physical and chemical methods for synthesis of PtNPs and PdNPs, biological methods are considered to be advantageous as they are more biocompatible and less toxic which is a prerequisite for an ideal candidate nanomedicine. Recently, we have shown the potential of medicinal plants like *Dioscorea bulbifera*, *Gnidia glauca*, *Plumbago zeylanica*, *Dioscorea oppositifolia*, *Barleria prionitis*, *Litchi chinensis*, and *Platanus orientalis* for synthesis of gold, silver, and bimetallic nanoparticles [4–15]. Medicinal plants are storehouses of variety of phytochemicals which may play a vital role in synthesis and stabilization of the bioreduced nanoparticles [16–23]. Hence, it is economical and efficient. Although we have reported its potential for synthesis of gold nanoparticles (AuNPs) and silver nanoparticles (AgNPs) earlier, there are no reports on synthesis of PtNPs and PdNPs till date by *Gloriosa superba* tuber extract (GSTE) [24]. *G. superba* is reported to harbour several groups of secondary metabolites such as alkaloids, flavonoids, glycosides, phenols, saponins, steroids, tannins, and terpenoids [25]. The roots are widely used as germicide, to cure ulcers, piles, haemorrhoids, inflammation, scrofula, leprosy, dyspepsia, worm's infestation, flatulence, intermittent fevers, debility, arthritis, and against snake poison [26]. But no extensive studies have been carried out till date on its nanobiotechnological applications.

In view of the background, herein we report synthesis of PtNPs and PdNPs using GSTE which was further characterized using UV-visible spectroscopy, high-resolution transmission electron microscopy (HRTEM), energy dispersive spectroscopy (EDS), dynamic light scattering (DLS), and X-ray diffraction (XRD) analysis. Furthermore, the bioreduced nanoparticles were checked for anticancer activity against MCF-7 cell lines.

## 2. Materials and Methods

### 2.1. Plant Material and Extract Preparation

GSTE was prepared by collecting *G. superba* fresh tubers from the Western Ghats of Maharashtra, India, which were thoroughly washed, chopped into small pieces, and shade-dried for 2 days. The dried tubers were reduced to fine powder in an electric blender, 5 g of which was added to 100 mL of distilled water in a 300 mL Erlenmeyer flask and boiled for 5 minutes and eventually collected by decantation followed by filtration through a Whatman number 1 filter paper. The resulting filtrate was used for synthesis of nanoparticles [14].

### 2.2. Synthesis and UV-Vis Spectroscopy

Reduction of PtCl_6_^2−^ ions was initiated by addition of 5 mL of GSTE to 95 mL of 10^−3^ M aqueous H_2_PtCl_6_·6H_2_O solution, while for synthesis of PdNPs, 5 mL of GSTE was mixed with 95 mL of 10^−3^ M aqueous PdCl_2_. The resulting mixtures were incubated at 100°C for 5 hours with constant stirring for synthesis of PtNPs and PdNPs which was monitored at regular intervals using UV-Vis spectroscopy on a spectrophotometer (SpectraMax M5, Molecular Devices Corp, USA) operated at resolution of 1 nm [18, 27].

### 2.3. High-Resolution Transmission Electron Microscopy (HRTEM), Energy Dispersive Spectroscopy (EDS), Dynamic Light Scattering (DLS), and X-Ray Diffraction (XRD)

Morphological features like size and shape of bioreduced PtNPs and PdNPs were determined using JEOL-JEM-2100 high-resolution transmission electron microscope (HRTEM) equipped with a energy dispersive spectrometer (EDS) at an energy range of 0–20 keV. Particle size was analyzed using the dynamic light scattering equipment (Zetasizer Nano-2590, Malvern Instruments Ltd., Worcestershire, UK) in polystyrene cuvette [14, 15]. The diffraction data for the dry powder were recorded on a Bruker X-ray diffractometer using a Cu K*α* (1.54 Å) source [28].

### 2.4. Fourier-Transform Infrared (FTIR) Spectroscopy

After 5 hours of synthesis of PtNPs and PdNPs using GSTE, the resulting mixture was centrifuged at 10,000 rpm for 15 minutes. The supernatant was collected which was added on KBr and dried. Similarly, GSTE before bioreduction was also used to compare the alteration of the phytochemistry. The KBr pellet containing GSTE before and after bioreduction was subjected to FTIR (IRAffinity-1, Shimadzu Corp, Tokyo, Japan) spectroscopy measurement in the diffused reflection mode at a resolution of 4 cm^−1^ subjected to the IR source 500–4000 cm^−1^ [8].

### 2.5. Anticancer Activity

Anticancer activities of PtNPs and PdNPs were compared using MTT (3-(4,5-dimethyl-thiazol-2-yl)-2,5-diphenyl-tetrazolium bromide) assay. MCF-7 cells were seeded (4 × 10^4^ cells/well) in a 96-well plate and incubated for adherence for 24 hours, at 37°C with 5% CO_2_ concentration followed by which nanoparticles were added at a final concentration of 200 *µ*g/mL and incubated for 48 hours. Medium was removed thereafter, and PBS was used to wash the cells. In each well, MTT (0.5 mg/mL) was added and incubated for 3 hours. The resulting formazan crystals were solubilised in acidified isopropanol, and the absorbance was measured at 570 nm. The statistical analysis was done by using one-way ANOVA.

### 2.6. Flow Cytometric Analysis

The mechanism underlying the anticancer activity of the PtNPs and PdNPs against MCF-7 cells was studied using flow cytometric analysis of cells treated with respective nanoparticles. 5 × 10^5^ cells were initially seeded in a T-25 flask and incubated for 24 hours followed by addition of PtNPs and PdNPs nanoparticles at a concentration of 200 *µ*g/mL. After 48 hours of incubation, the cells were harvested and stained with Annexin V-FITC (dilution 1 : 20) and propidium iodide (dilution 1 : 20) for 15 minutes at 4°C and were acquired using BD FACSVerse and analyzed by BD FACSuit software as reported earlier [8, 14].

### 2.7. Confocal Microscopy

In order to support flow cytometric analysis, immunofluorescence staining was performed to find out the mechanism of cell death in MCF-7 cells on treatment with PtNPs and PdNPs. Cells were seeded at a density of 5 × 10^4^ cells on to glass coverslips followed by incubation for 24 hours for adherence and then treated thereafter with 200 *µ*g/mL of PtNPs and PdNPs for 48 hours. The treated cells were stained with Annexin V(AV)-FITC and PI, both at a dilution of 1 : 20 for 15 minutes at 4°C followed by observation under the LSM 780 confocal laser scanning microscope, Carl Zeiss [8, 14, 24].

## 3. Results and Discussion

### 3.1. UV-Visible Spectra

**Figure 1:**
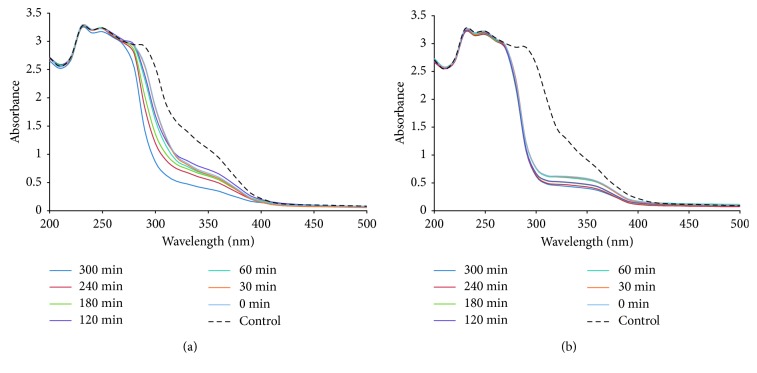
UV-Vis spectra recorded at different time intervals for nanoparticle formation using GSTE at 100°C with (a) 1 mM H_2_PtCl_6_·6H_2_O solution and (b) 1 mM PdCl_2_ solution. Control represents corresponding salt solution without GSTE.

GSTE served as source of the phytomolecules which could efficiently synthesize and stabilize PtNPs and PdNPs that were further studied for anticancer activity. Development of brown colour on addition of GSTE in H_2_PtCl_6_·6H_2_O salt solution on incubation at 100°C indicated the synthesis of PtNPs. UV-visible spectra showed the decrease in the intensity specific to the H_2_PtCl_6_·6H_2_O salt solution till 5 hours, beyond which no significant decrease was observed which confirmed the completion of the synthesis (Figure 1(a)). Similarly, initially, dark brown colour was developed which eventually turned into black on reaction of GSTE with PdCl_2_ solution under same conditions. Decrease in the intensity of the UV-spectrum corresponding to PdCl_2_ solution confirmed the synthesis of PdNPs within 5 hours (Figure 1(b)). This result is well in agreement with the previous reports where nanoscale PtNPs and PdNPs were synthesized using medicinal plants like *D. bulbifera* and *B. prionitis* [8, 14]. The synthesis was found to be faster as compared to synthesis using *Glycine max* and *Cinnamomum camphora*, both of which took 48 hours for complete synthesis of PdNPs [29, 30]. As displayed in Figure 1, the absorption spectra of platinum and palladium colloidal suspensions after 5 hours of bioreduction by GSTE were compared with the absorption spectra of their respective salt solution. Previous reports confirm that the absorption bands appearing in the contrast spectrum of corresponding salt solution were ascribed to the ligand-to-metal charge-transfer transition of the ions. The absence of the absorption peaks above 300 nm in all the samples after 5 hours indicated complete reduction of the metal ions. Similar accreditation was made during thermally induced reduction of Pd(Fod)_2_ in o-xylene and sonochemical reduction of Pd(NO_3_)_2_ in aqueous solution, respectively. Absence of absorption peaks was consistent with the theoretical study of the surface plasmon resonance absorption of PdNPs. The spectra of colloidal suspensions of PtNPs and PdNPs presented broad absorption continua extending throughout the visible-near-ultraviolet region, which were also observed earlier for the platinum group of metals [31–35].

### 3.2. HRTEM Analysis

**Figure 2:**
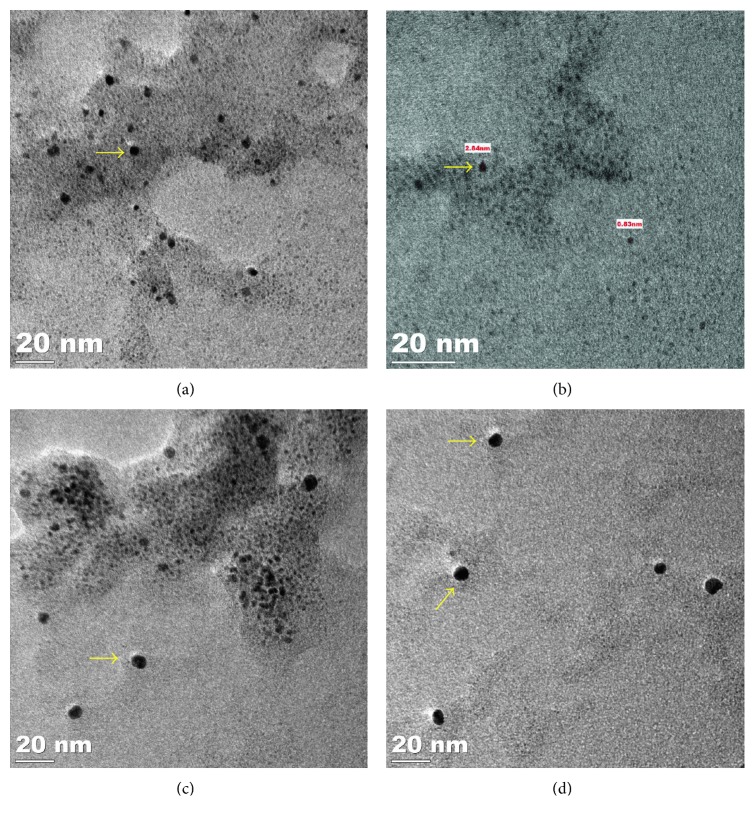
HRTEM images of nanoparticles synthesized by GSTE: PtNPs with inset scale bar showing (a) 100 nm and (b) 20 nm; PdNPs with inset scale bar showing (c) 20 nm and (d) 20 nm.

**Figure 3:**
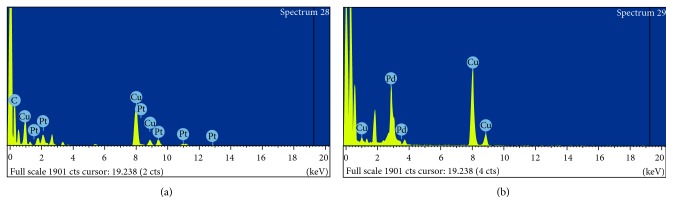
Representative spot EDS of nanoparticles synthesized by GSTE: (a) presence of platinum in PtNPs; (b) presence of palladium in PdNPs.

**Figure 4:**
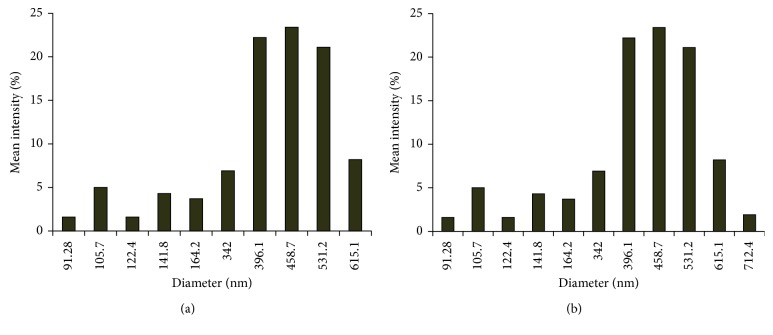
Dynamic light scattering measurement showing size distribution of nanoparticles synthesized by GSTE: (a) PtNPs; (b) PdNPs.

Morphological analysis of the as-synthesized PtNPs and PdNPs was performed using high-resolution transmission electron microscopy (HRTEM). Figures 2(a) and 2(b) reveal the size and shape of the bioreduced PtNPs. The synthesized PtNPs were very small that were majorly of spherical shape, while the diameter was in a range from 0.8 nm to 3 nm. In the magnified overview of the image, the particles were seen to be embedded in a biological matrix may be derived from the GSTE which can play a critical role in the stabilization process. *Diospyros kaki* was reported to synthesize PtNPs of larger size, the diameter was found to be in a range between 2 and 12 nm [36]. At 90°C, *Cacumen platycladi* is reported to synthesize very small PtNPs varying in a range of 2.4 ± 0.8 nm [37, 38]. Figures 2(c) and 2(d) showed the morphological characteristics of the PdNPs which were also predominantly spherical in shape, and the diameter of the particles was found to vary in a narrow range between 5 and 8 nm. It is very rare to get such monodispersed uniform nanoparticles using a biological route. Similarly, previous study reports that PdNPs synthesized using *Glycine max* were found to be bigger in size which was 15 nm in diameter [29]. The energy dispersive spectra profile confirmed the presence of elemental platinum and palladium in PtNPs and PdNPs, respectively (Figure 3). Hydrodynamic size recorded for the bioreduced nanoparticles was also in agreement with the observed HRTEM data. However, larger dimensions were also visualized in DLS spectra which may be due to the nanoparticles trapped in the phytochemical entities from GSTE (Figure 4) [7].  Table 1 gives a comprehensive account of various medicinal plants like *Anacardium occidentale*, *Piper betle*, *Annona squamosa*, *Terminalia chebula*, and *Pulicaria glutinosa*, which are reported to synthesize either PtNPs, PdNPs, or both [37, 39–41].

### 3.3. X-Ray Diffraction (XRD) Analysis

**Figure 5:**
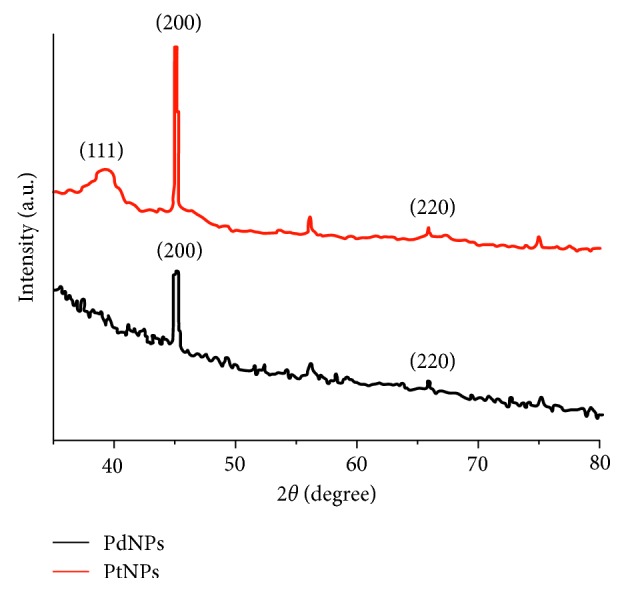
Representative X-ray diffraction profile of thin film PtNPs and PdNPs, synthesized by GSTE.

The as-synthesized nanoparticles were characterized for their phase with the help of XRD. The powder diffraction data of the dried powder was recorded on a Bruker X-ray diffractometer with Cu K*α* (1.54 Å) source.  Figure 5 shows the XRD data of the PtNPs and PdNPs. The sharp peaks in case of PtNPs and PdNPs represent the crystalline nature of both the nanoparticles. The phase formation has also been confirmed from the data [8]. The characteristic peaks, as seen in Figure 5, correspond to the lattice planes (111), (200), and (220) in case of PtNPs; however, (111) plane was not seen in case of PdNPs. The reason for absence (or no growth) of the (111) plane in case of PdNPs needs to be explored, but at the preliminary stage, we feel that the plant extract might have some crucial role in such restricted growth.

### 3.4. FTIR Analysis

**Figure 6:**
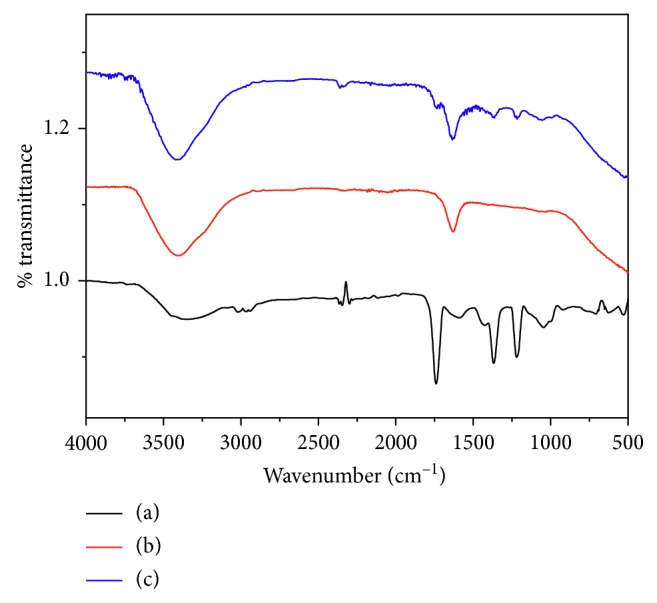
FTIR spectra of GSTE. (a) Before synthesis of nanoparticles, (b) after synthesis of PtNPs, and (c) after synthesis of PdNPs.

FTIR spectral analysis showed various functional groups in GSTE before bioreduction and their alteration after synthesis of PtNPs and PdNPs (Figure 6). GSTE showed a prominent peak of the hydroxyl group specific to alcoholic and phenolic compounds at ∼3300 cm^−1^, which remain unaltered even after nanoparticles synthesis. Similarly, peaks observed at 1049, 1218, 1369, and 1737 cm^−1^ can be attributed to the C-O-C bond in ether, unassigned amide mode, CH_3_ bend, and stretching of C=O bond, respectively, which disappeared after synthesis of nanoparticles. This indicates that phytochemicals with abovementioned functional groups are responsible for reduction of the metal ions salts leading to synthesis of corresponding nanoparticles. However, a significant feature of the amide bond at 1627 cm^−1^ seen in GSTE is recovered after synthesis, suggesting the replacement of carboxylic group by amines, which in turn again supports the hypothesis of role of carboxylic and similar groups in reduction of the metal salts into the corresponding metal nanoparticles [49].

### 3.5. Anticancer Activity

**Figure 7:**
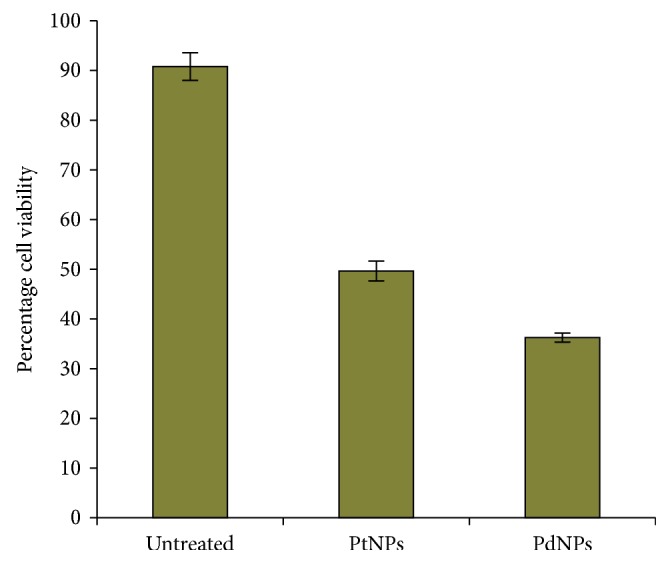
Anticancer activity against MCF-7 cells using MTT reduction assay. The data are indicated as the mean ± SEM (*n*=5).

**Figure 8:**
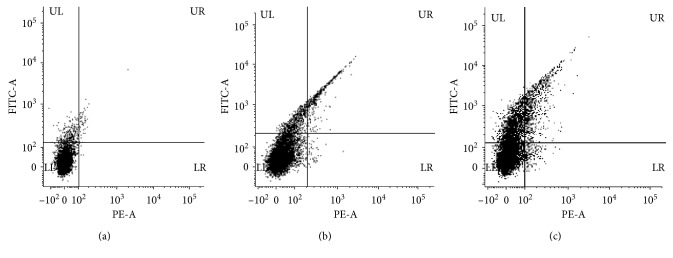
Flow cytometric analysis for MCF-7 cells treated with PtNPs and PdNPs for 48 hours confirming phosphatidyl serine externalization (Annexin V-FITC binding) and cell membrane disintegration (PI staining) The dual parametric dot plots combining Annexin V-FITC and PI fluorescence show the viable cell population (lower left quadrant, Annexin V-FITC^−^ PI^−^), the early apoptotic cells (lower right quadrant, Annexin V-FITC^+^PI^−^), and the late apoptotic cells (upper right quadrant, Annexin V-FITC^+^PI^+^). (a) Untreated cells; (b) treatment with PtNPs; (c) treatment with PdNPs.

**Figure 9:**
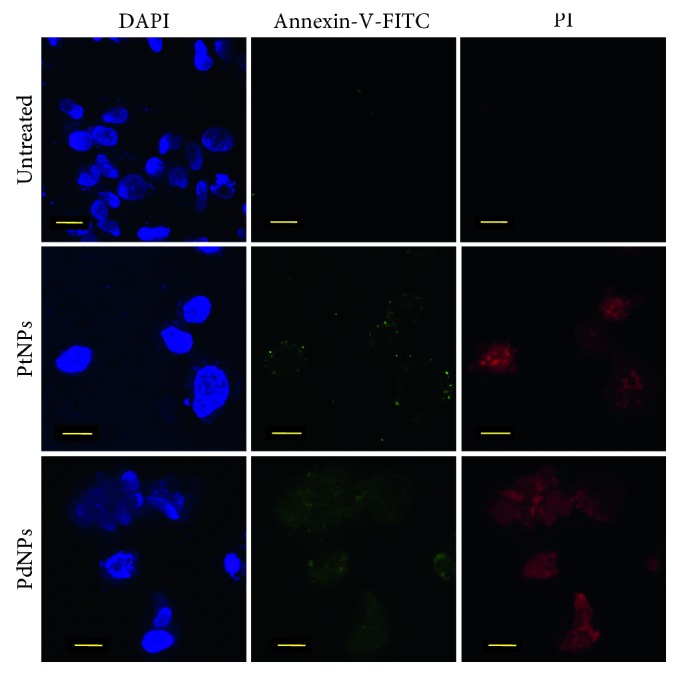
Confocal imaging of apoptosis induction by PtNPs and PdNPs in MCF-7 cells seeded on coverslips and stained with Annexin V-FITC and PI.

Apoptosis is considered as programmed cell death orchestrated by cascade of interdependent synchronised cellular events. It is the most critical process for maintenance of homeostasis, where an efficient balance between cell proliferation and cell death is maintained [50]. Fabrication of apoptotic nanoinducers is of prime importance to develop novel nanomedicine against cancer. Platinum drugs like cisplatin, oxaliplatin, and carboplatin are considered as candidates for treatment and management of cancer, although they pose a threat of potential adverse effects. However, there are very less studies on the anticancer activity of biologically synthesized PtNPs and PdNPs. In our study, both PtNPs and PdNPs showed superior anticancer activity by reducing the viability of MCF-7 cells on treatment till 48 hours. PtNPs showed an anticancer activity up to 49.65 ± 1.99%, while PdNPs showed an activity up to 36.26 ± 0.91% (Figure 7). PtNPs and PdNPs are reported to exhibit high cytotoxicity owing to their physicochemical interactions with the functional groups of cellular proteins, nitrogen bases, and phosphate groups of the DNA leading to cell death. Earlier reports confirm that Pd leads to formation of free radicals, leakage of lactate dehydrogenase, and cell-cycle disturbances which can be the key underlying mechanism behind the anticancer activity [3]. Cellular deaths are mainly due to either apoptosis, autophagy, or necrosis. In order to determine the percentage of apoptotic and necrotic cells, MCF-7 cells were treated with 200 *µ*g/mL of both PtNPs and PdNPs for 48 hours and stained with Annexin V and PI followed by flow cytometric analysis (Figure 8). Both PtNPs and PdNPs were capable of inducing apoptosis in MCF-7 cells up to 12.32% and 31.3%, respectively, which was found to be higher compared to previous reports on human lung adenocarcinoma (A549), ovarian teratocarcinoma (PA-1), pancreatic cancer (Mia-Pa-Ca-2) cells, and normal peripheral blood mononucleocyte (PBMC) cells [2]. Our results were comparable to anticancer activity of PdNPs synthesized using *Camellia sinensis* against human leukemia (MOLT-4) [3]. Recently, such unconventional platinum anticancer agents and associated nanomedicines have got more attention as clinically successful platinum drugs like cisplatin, carboplatin, and oxaliplatin have exhibited tremendous deleterious side effects that include nephrotoxicity, fatigue, emesis, alopecia, ototoxicity, peripheral neuropathy, and myelosupression [51, 52]. Confocal images also confirmed the induction of apoptosis (Figure 9). Externalization of phosphatidyl serine and membrane disintegration was evident from Annexin V-FITC^+^PI^+^ MCF-7 cells. Similarly, membrane blebbing and chromosome condensation were also observed in PtNPs treated cells, which is a critical hallmark of apoptosis [8].

## 4. Conclusion

Monodispersed PtNPs and PdNPs were synthesized using *G. superba* tuber extract which were found to be uniformly spherical and almost isodiametric. The synthesis was found to be rapid, efficient, and environmentally benign. Both PtNPs and PdNPs showed potent anticancer activity against MCF-7 (human breast adenocarcinoma) cells. The mechanism of cell death was confirmed to be induction of apoptosis characterized by phosphatidyl serine externalization, membrane disintegration, and blebbing with chromosome condensation. Further studies on these phytogenic nanoparticles might help to establish their potential as candidate drugs against breast cancer.

## Figures and Tables

**Table 1:** Phytogenic PtNPs and PdNPs.

Serial number	Plant	Extract used	NPs	Shape	Size (nm)	Reference
1	*Cacumen platycladi*	Whole biomass	PtNPs	Spherical	2.4 ± 0.8	[38]
2	*Anacardium occidentale*	Leaf	PtNPs	Irregular and rod shaped	—	[39]
3	*Diospyros kaki*	Leaf	PtNPs	Spheres and plates	2–20	[36]
4	*Ocimum sanctum*	Leaf	PtNPs	Irregular	23	[42]
5	*Fumariae herba*	Whole herb	PtNPs	Hexagonal and pentagonal	30	[43]
6	*Curcuma longa*	Tuber	PdNPs	Spherical	15–20	[44]
7	*Gardenia jasminoides* Ellis	Fruit	PdNPs	Spherical, rod, and three-dimensional polyhedra	3–5	[45]
8	*Glycine max*	Leaf	PdNPs	Spherical	15	[29]
9	*Punica granatum*	Peel	PtNPs	Spherical	16–23	[46]
10	*Cinnamomum camphora*	Leaf	PdNPs	Irregular	6	[30]
11	*Annona squamosa* L.	Peel	PdNPs	Spherical	100	[41]
12	*Pulicaria glutinosa*	Whole plant	PdNPs	Spherical	20–25	[46, 47]
13	*Delonix regia*	Leaf	PdNPs	Spherical	2–4	[48]
14	*Piper betle* L.	Leaf	PtNPs	Spherical	2.1 ± 0.4	[40]
			PdNPs	Spherical	3.8 ± 0.2	
15	*Dioscorea bulbifera*	Tuber	PtNPs	Spherical	2–5	[8]
			PdNPs	Spherical and blunt ended cubes	10–25	
16	*Barleria prionitis*	Leaf	PtNPs	Spherical	1-2	[14]
			PdNPs	Spherical and irregular	5–7	

## Data Availability

The data used to support the findings of this study are available from the corresponding author upon request.
